# Review of the temporal and geographical distribution of measles virus genotypes in the prevaccine and postvaccine eras

**DOI:** 10.1186/1743-422X-2-87

**Published:** 2005-11-22

**Authors:** Michaela A Riddell, Jennifer S Rota, Paul A Rota

**Affiliations:** 1 Scientist/PhD Scholar, Victorian Infectious Diseases Reference Laboratory/WHO Western Pacific Measles Regional Reference Laboratory and Department of Public Health, School of Population Health, University of Melbourne, Parkville 3010, Victoria, Australia; 2 Centers for Disease Control and Prevention, Atlanta, GA, 30333 USA; 3 Dept. Molecular Microbiology and Immunology, Johns Hopkins School of Public Health, Baltimore MD 21205 USA

## Abstract

Molecular epidemiological investigation of measles outbreaks can document the interruption of endemic measles transmission and is useful for establishing and clarifying epidemiological links between cases in geographically distinct clusters. To determine the distribution of measles virus genotypes in the prevaccine and postvaccine eras, a literature search of biomedical databases, measles surveillance websites and other electronic sources was conducted for English language reports of measles outbreaks or genetic characterization of measles virus isolates. Genotype assignments based on classification systems other than the currently accepted WHO nomenclature were reassigned using the current criteria. This review gives a comprehensive overview of the distribution of MV genotypes in the prevaccine and postvaccine eras and describes the geographically diverse distribution of some measles virus genotypes and the localized distributions of other genotypes.

## Introduction

Although measles virus (MV) is serologically monotypic, the genetic characterization of wild-type viruses has identified eight clades (A – H), which have been divided into 22 genotypes and one proposed genotype. Clades B, C, D, G and H each contain multiple genotypes (B1 – 3, C1 – 2, D1 – 10, G1 – 3, H1 – 2) while clades A, E and F each contain a single genotype (A, E, F) [1,2]. The sequences of the vaccine strains indicate that the wild type viruses from which they were derived were all members of genotype A. All measles genotypes can be neutralized by serum from vaccinated persons *in vitro*, although with varying efficiency [3,4]. There are no known biological differences between viruses of different genotypes. Specific measles genotypes are not associated with differences in severity of disease, likelihood of developing severe sequela such as subacute sclerosing panencephalitis or inclusion body encephalitis, or variability in sensitivity of laboratory diagnosis.

Analysis of the variability in the nucleotide sequences of wild-type MVs has enabled the use of molecular epidemiologic techniques for measles surveillance. The molecular data, when used in conjunction with standard case reporting and investigation, can help to identify epidemiological links between geographically distinct cases and outbreaks as well as track importations of MV. [5-7]. Also, approximately 5% of vaccine recipients experience mild symptoms (rash and fever) after vaccination and these cases could be misclassified as wild-type measles [8]. Genetic characterization of viral isolates or RT-PCR products is the only laboratory test that can differentiate between vaccine-associated cases and wild-type infection [6,9,10].

In 1998, the World Health Organization (WHO) recommended a standard protocol for the designation of measles genotypes. These recommendations, updated in 2001 and 2003, also included a standard analysis protocol and designation of standard reference strains (see Additional file 2) against which all newly characterized isolates should be compared [2,11,12]. The minimum amount of sequence data required to assign a virus to a genotype are the 450 nucleotides encoding the carboxy terminus of the N protein. The entire sequence of the coding region of the H gene should be obtained from representative isolates [11]. New genotypes are designated if the nucleotide sequence differs from the closest reference sequence by more than 2.5% in N and 2.0% in H [2]. Additionally, phylogenetic analysis should produce similar tree topographies using at least two different analysis methods. Several isolates or clinical specimens should be sequenced and at least one viral isolate should be available as the reference strain. Finally, new genotype classifications should be useful for epidemiological studies, by providing a means to identify the source or transmission pathway of infection and by contributing to our understanding of the global distribution of MV genotypes [2].

The purpose of this summary is to collate all available reports of MV genotypes and to standardize the published genotype nomenclature, according to the current WHO criteria, with the aim of giving a comprehensive overview of the distribution of MV genotypes in the prevaccine and postvaccine eras.

## Methods

An examination of the National Library of Medicine "PubMed" [13] search engine using the keyword "measles" combined with "genotypes" and "sequence" was performed to identify English language publications or abstracts describing measles genotyping.

Additional sources included the reference lists of articles identified by "PubMed" and electronic sources such as the CDC and PAHO measles network Internet pages and the NCBI Genbank website [14-16]. Measles outbreak alerts were received through the WHO network, which distributes outbreak notifications. In addition, subscription based electronic newsletters such as ProMED mail [17] and Immunization newsbrief [18] were scrutinized for information relating to measles outbreaks. Direct contact was made with the notifying laboratory or health unit requesting genotype information if available.

A table produced by participants at the 1998 WHO meeting listed older classification systems and the comparable genotype classifications under the universally accepted system. This table was used to reclassify genotypes cited in publications prior to 1998 [11]. In some cases, later publications from the same or other groups were used to assign current genotypes to viruses classified before 1998.

## Results and Discussion

One hundred and twenty eight studies were identified through the PubMed search, 67 of which described the genotype of MV isolates. Four internet websites were identified (including Genbank) and a further 27 articles were identified from the reference lists of cited publications or from outbreak notification lists such as ProMED [17] and Immunization Newsbrief [18].

**Figure 1:**
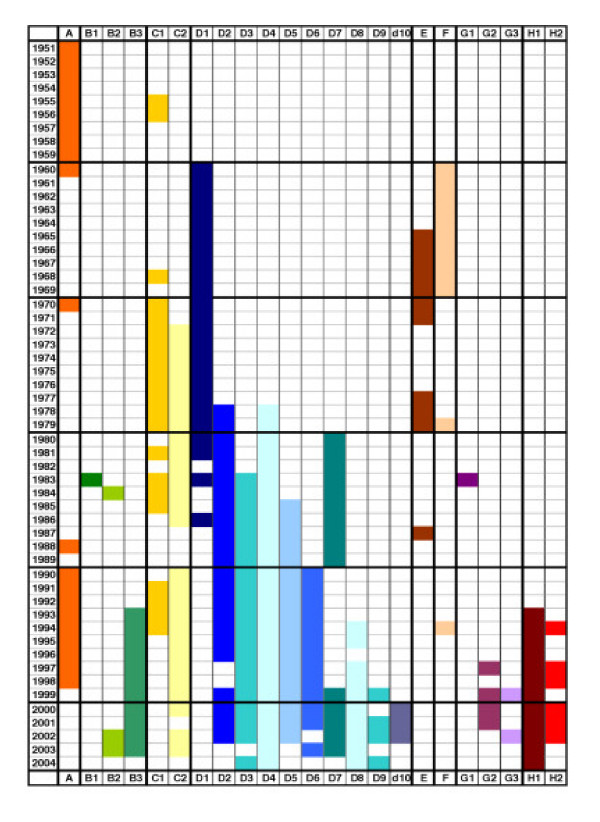
Temporal distribution of measles virus genotypes 1951 – 2004. Summary of distribution of MV genotypes from the prevaccine era to 2004. Refer to Additional file 1 for complete referencing of data shown in figure. *Data reflects publications available as of August 2005.*

Figure 1 and Table 1 summarize the temporal and geographical distribution of MV genotypes from the early 1950s to 2004 but do not differentiate between cases of endemic or imported measles virus. Genotype and location specific references are not cited in the following results section but can be found in the relevant genotype specific section in the comprehensive table which accompanies this paper (see Additional file 1, also available from the website of the WHO Western Pacific Regional Reference Laboratory for measles, The Victorian Infectious Diseases Reference Laboratory, Melbourne, Australia ).

**Table 1:** Distribution of MV genotypes by WHO geographical region 1950s – 2004. Countries in which MV virus has been detected. No distinction has been made between endemic transmission or instances of MV importation. (data reflects publications available as of August 2005). Refer to Additional file 1 for details of endemic transmission and imported measles cases and for complete referencing of data.

Geno-type	AFRO^1^	EMRO^2^	SEARO^3^	WPRO^4^	Europe^5^	Americas^6^
A				China, Japan	Romania, UK Finland, Russia, Czech Republic, Slovakia	Brazil, USA, Argentina,

B1	Cameroon					
B2	Gabon South Africa, Angola					
B3	Gambia, Nigeria, Kenya, Ghana, Algeria, Cameroon, Rep. of Congo, Dem. Rep. of Congo Burkina Faso, Equatorial Guinea	Sudan, Tunisia, Libya			France, Spain Germany, UK,	USA

C1				Japan	Nth Ireland, Spain, Germany	USA, Canada, Argentina
C2	Zimbabwe	Morocco		Australia	Austria, France, Belgium, Netherlands, Czech Republic, Slovakia, Spain, Italy, Germany, UK, Luxembourg, Denmark,	USA, Brazil, Canada,

D1				Australia	UK, Nth Ireland	
D2	South Africa, Zambia				Ireland, UK, Spain	USA
D3	South Africa			Micronesia, Philippines, PNG, Japan, Australia, Taiwan	UK, Denmark	USA, Canada
D4	South Africa, Namibia, Kenya, Ethiopia	Pakistan, Lebanon, Afghanistan, Syria, Iran	India, Nepal	Japan, Australia	UK, Denmark, Netherlands, Germany, Spain, Croatia, Russia,	USA, Canada
D5	Namibia		Thailand, Bangladesh	Japan, Malaysia, Micronesia, Australia, New Zealand, Cambodia, Guam, Rep. of Korea	UK, Germany	South America, USA, Canada, Brazil
D6					UK, Ireland, Spain, Germany, Austria, Italy, Greece, Croatia, Turkey, Ukraine, Poland, Russia, Luxembourg, Bosnia, Israel, Norway, Denmark, Netherlands	USA, Canada, Brazil, Bolivia, Argentina, Uruguay, Dominican Republic \Haiti
D7			Sri Lanka, Myanmar (Burma), India	Australia	UK, Germany, Sweden, Europe, France, Spain, Italy	El Salvador, USA, Canada, Mexico
D8	Ethiopia	Pakistan, Oman,	India, Bangladesh, Nepal	Australia	UK, Spain, Yugoslavia, Albania, Italy, Lithuania	USA, Canada
D9			Indonesia	Australia, Japan	Europe	Venezuela, Colombia
D10	Uganda					

E					Germany, Denmark	USA, Canada

F					Spain	

G1						USA
G2	South Africa		Indonesia	Australia, Malaysia	Netherlands, UK, Germany	Mexico, USA
G3			E. Timor, Indonesia	Australia		

H1			Thailand	Australia, China, New Zealand, Mongolia, Singapore, Japan, Rep. of Korea, Rep. of Marshall Islands	UK, Spain, Netherlands, Denmark, Germany	USA, Canada, Chile, Mexico
H2				China, Vietnam, Australia		USA

^1^AFRO – WHO African region, ^2^EMRO – WHO Eastern Mediterranean region, ^3^SEARO – WHO South East Asian region, ^4^WPRO – WHO Western Pacific region, ^5^Europe – WHO European region, ^6^Americas – WHO region of the Americas

Routine molecular characterization of wild-type measles viruses was initiated in response to a global resurgence of measles disease in the late 1980s and the concurrent availability of sensitive techniques (e.g. RT-PCR and automated sequencing) for the investigation of viral genomes. Prior to that date, only a few isolates of measles were available for molecular characterization and reliable epidemiologic information was not available for many of these isolates. In the era before the widespread use of measles vaccine, genotypes A, C1, and D1 were detected. Genotype A virus includes the prototype Edmonston strain, the progenitor for most of the current measles vaccines. Analysis of MV sequences obtained from SSPE cases, resulting from initial infections that occurred during the 1950s and 1960s, detected genotypes C1, D1, E and F, providing further evidence that genotype A was not the only genotype detected during the prevaccine era [19-24]. However, data from these earlier studies must be interpreted cautiously due to the large number of mutations in SSPE sequences and the lack of standardization. Of course, detection of various genotypes in SSPE cases reflects efforts to study this devastating illness and should not be taken as an indication that one genotype is more likely to cause SSPE than another [25]. Retrospective sequence analysis of viral isolates collected during the 1970s showed continued detection of genotypes C1 and D1 and the first detections of genotypes C2, D2, D4, E and F.

As virologic surveillance expanded in the late 1980s and 1990s, the number of genotypes detected in cases and outbreaks increased substantially to include the 23 genotypes now recognized by the WHO. However, some genotypes (B1, D1, E, F, G1) have not been detected in the last 15 years and are considered inactive.

Genotype A has been detected in acute cases of measles in South and North America, China, Japan, Eastern Europe, Finland and the UK over the last 40 years. Since, it is difficult to distinguish wild-type viruses in genotype A from vaccine strains, these reports must be interpreted with caution since some of the sequences may have been derived from vaccine associated cases or been the result of laboratory contamination [22,26,27]. In the future, detection of genotype A viruses in association with acute cases of measles will need to be thoroughly scrutinized and additional sequence data will need to be obtained from both clinical samples and corresponding viral isolates.

Genotype B2, previously considered inactive [12], has recently been detected in South Africa and Angola [28]. Genotype B3 was first detected in 1993 in Gambia but has subsequently been detected in cases from Cameroon, Nigeria, Ghana, Burkina Faso, DR Congo and the Sudan. This genotype is the endemic genotype of West and Central Africa and has been imported into numerous countries including France, Germany and the USA.

Outbreaks involving genotype C1 have occurred in Canada, Japan, Germany and most recently in the early 1990s in Argentina, which was the last reported outbreak involving genotype C1 circulation. Genotype C2 has circulated widely throughout the European continent and has been exported to the USA and Canada from France, Italy and Germany, where it was known to be an endemic genotype until 2001. This genotype was also identified in Australia from 1990 to 1991 and Morocco in 1998 & 1999. An importation of genotype C2 to the USA was linked with travel from Zimbabwe in 1998 although there are no reports to indicate that this strain was circulating in Southern Africa during this time [6].

Characterization of archived MV isolates in Australia from 1971, suggest that genotype D1 may have been the endemic strain in Australia during the pre-vaccine era. Sequences from SSPE cases in Northern Ireland and the UK indicate that genotype D1 was also detected in Britain before the widespread use of vaccine. Genotype D1 has not been detected since 1986 and is considered inactive. Genotype D2 appears to have been the endemic strain of Southern Africa from the late 1970s to 2000. This genotype was also responsible for the large outbreak in Ireland in 1999 – 2000, which resulted in importations to both the UK and USA. Genotype D3 is currently endemic in Papua New Guinea and possibly the Philippines, given that several measles cases in the USA have been linked with travel from the Philippines. Additionally this genotype has been associated with a case of SSPE in South Africa, and has been detected in Australia, USA and Canada, the UK and Denmark, in most cases with epidemiological links of importation from Japan or the Philippines. Genotype D4 is widely distributed and has been associated with multiple outbreaks on the Indian sub-continent, East and South Africa and a large outbreak in Quebec Province, Canada in 1989. Recently genotype D4 viruses, imported from the Indian sub-continent and East and South Africa, have been epidemiologically linked with cases in Canada, the USA, the UK, other European countries and Australia. Genotypes D4 and D2 appear to have been co-circulating in Southern Africa from the late 1970s to the late 1990s. Genotype D5 is endemic in Cambodia and has been associated with measles cases detected in the Americas, the UK, Germany and Australia. Epidemiological investigations have identified Japan and Thailand as the main sources for these importations. Until recently both genotype D3, and genotype D5 were endemic in Japan [26,29-31]. However, recent evidence suggests that these genotypes may no longer be predominant in Japan [32]. Genotype D6 has circulated widely throughout the European continent and may have been the endemic genotype of Europe, in conjunction with genotype C2, since the 1990s. This genotype is endemic in Turkey [33] and the Russian Federation [34]. Genotype D7 circulated in the UK and Australia during the 1980s. Chains of transmission of this genotype have been associated, through epidemiological investigations, with Sweden and other European countries, including Italy where it was identified in the large measles outbreak in 2002. Genotype D7 has been imported into the US from multiple European sources from 2001 to 2003. Recently this genotype replaced genotypes C2 and D6 as the most commonly isolated genotype in Germany [35]. Genotype D8 appears to be co-circulating with genotype D4 on the Indian sub-continent and Ethiopia, although the first known description of this genotype was in the UK, from where it has been regularly detected. However, investigations have linked UK cases with importations of virus not only from the Indian sub-continent but also from the Balkans and Oman [36]. Genotype D8 has been imported into Australia and the USA from India and Bangladesh. Genotype D9, first described after importation to Australia from Indonesia (Bali) in 1999, was isolated during the large outbreak in 2000 – 2001 in Colombia and Venezuela. D9 was associated with an outbreak in Japan in 2004. Analysis of wild-type viruses isolated in Uganda in 2000–2002 indicated the presence of a new genotype, which has been proposed as genotype d10 [Genbank accession numbers AY923185 through AY923212] [37].

A few genotype E viruses and related SSPE cases were reported in the early 1970s. Genotype F sequences have been identified on two occasions, both were SSPE cases wherein acute measles infection was documented in 1967 and 1968. Thus both genotypes E and F probably circulated in the pre-vaccine era [19,22].

Clade G, previously consisting of one genotype (G), has recently been expanded to contain three genotypes. The original genotype G (now G1) had not been detected since 1983 and was thought to have been extinct. However, recent investigations have identified two new genotypes (G2 & G3), both of which have been predominantly associated with chains of transmission within and importation from Indonesia and Malaysia [38-40].

Clade H viruses originally consisted of a single genotype but recently this clade has been expanded to contain two genotypes (H1 & H2). Both genotypes are predominant in the Asian and South East Asian regions. Genotype H1 has mainly been associated with transmission within or importations from China and was detected during the large measles epidemic in Korea in 2000 – 2001 [41]. Genotype H1 may now be dominant in Japan [32,42]. The WHO Western Pacific Regional Reference Laboratory for measles recently confirmed circulation of this genotype in Mongolia. Genotype H2, first described from samples recovered from China, has been more recently associated with importations from Vietnam [43].

Some genotypes of MV are associated with a particular geographical region, while other genotypes are more widely distributed. In particular, clade B is predominant in measles transmission in Sub Saharan and Central Africa, clade G in South East Asia and clade H in South East Asia and China. Clade D viruses, on the other hand, appear to be more widely distributed and are endemic in Eastern Africa, parts of Europe and the Indian sub-continent.

Determination of measles genotypes in countries that have not yet conducted molecular surveillance can be investigated, by proxy, from cases epidemiologically linked to imported cases. For example, the Philippines have not reported an endemic MV genotype but multiple importations to the USA associated with travel to, or contact with, the Philippines have resulted in the supposition that genotype D3 is the predominant circulating genotype in the Philippines [6,44]. However, caution must be taken when identifying genotypes by proxy as the genotype detected may not be the type that is endemic in the region. In some cases, genotypes have been epidemiologically linked to countries with no history of circulation of that genotype. For example, genotype G2 has been reportedly associated with importations to the UK from Mexico, South Africa and Australia, none of which have reported endemic circulation of genotype G2 [36]. In these cases infection may have occurred while the patient was in transit or at venues frequented by other travellers and might not reflect the circulating genotype.

Simultaneous circulation of multiple genotypes has been reported in several regions. Genotypes D3 and D5 co-circulated in Japan since the mid 1980s and the relative number of isolations changed over time. During the late 1980s genotype D3 was detected more frequently, but by 1990 D5 was more common [42,45]. Genotypes D2 and D4 appear to be co-circulating throughout Eastern and Southern Africa. Genotypes C2 and D6 continue to be detected in some parts of Europe and North Africa [35].

Rima et al described a shift from genotype C2 to genotype D6 in Spain in the early 1990s [24]. Santibanez et al recently demonstrated the shift from detection of mostly genotypes C2 and D6 in Germany to detection of mostly genotype D7 [46]. The shift of genotypes occurs in countries that have sub-optimal measles control programs, resulting in interruption of endemic transmission for short periods. However, failure to maintain high levels of population immunity results in the accumulation of susceptible individuals and creates conditions that favour the rapid transmission of a newly introduced genotype. Therefore, the apparent genotype switching is most likely due to changes in the distribution of susceptible individuals in the region.

Nine new MV genotypes have been identified since 1990 reflecting increased surveillance of measles cases and technological advances, rather than recent evolution. The designation of new genotypes, such as the newly proposed genotype d10, is likely to continue as the molecular analysis of viral isolates becomes routinely integrated into more countries within the global WHO measles laboratory network and more sequence data are added to the database. For example, genotype B3 may eventually be reclassified as two separate genotypes since this genotype contains viruses in two distinct clusters [47-49]. Characterization of viruses imported into Australia has detected three previously unrecognised genotypes (D7, D9 & G3) due partly to the frequency of travel between South East Asia and Australia and also to the comprehensive measles surveillance conducted by Australian laboratories [7,38,50].

The mutation rate amongst field isolates of MV is low and appears to be random rather than driven by vaccine pressure or immune responses [3,24,26]. Within a genotype, nucleotide differences (virus lineage) can assist in distinguishing separate episodes of transmission [24,51,52]. In countries or regions with endemic (ongoing and constant MV transmission) measles, many lineages of a single genotype may co-exist; however as countries begin to move from endemic to epidemic measles (MV transmission resulting in a higher number of cases than normally expected, typically against a background of little or no MV transmission)[53], the diversity of sequences within the circulating genotypes decreases [43,54-57]. In fact, the genotype D6 virus associated with a large measles outbreak that occurred in several South American countries between 1996 and 1997 had identical N gene sequences suggesting rapid spread of a single lineage [51]. Analysis of measles viruses circulating in Burkina Faso, before and after a mass vaccination campaign, showed that the number of circulating lineages was greatly reduced following the campaign. Sequence analysis of viruses isolated from outbreaks that occurred after the vaccination campaign suggested that virus was introduced from a single source [57].

Many recent measles outbreaks have been reported with no accompanying molecular genotyping investigations, for instance in Afghanistan [58], Niger [59] and the Philippines [60]. These outbreaks highlight the need to extend molecular surveillance capabilities to regions where measles remains endemic. Recent studies have described the recovery of MV RNA by RT-PCR from oral fluid, dried blood and dried oral fluid [61-63]. These samples, which are easy to collect, prepare and transport by post to laboratories capable of MV genotyping, have the potential to extend molecular surveillance for measles virus to remote settings and countries with limited infrastructure. However conventional samples such as nasopharyngeal swabs, urine and peripheral blood lymphocytes should continue to be collected, if logistically possible, because of the higher sensitivity of these sample types for detecting MV RNA.

Molecular surveillance undertaken in the early stages of measles control can facilitate identification of endemic genotypes. Over time or after intervention programs continued molecular surveillance, in conjunction with case based epidemiological investigations, can detect the interruption of endemic transmission [1]. Additionally, molecular analysis of specimens from cases facilitates both linkage to, and separation from, contemporaneous cases and clusters, assisting classical epidemiological investigations and the tracking of chains of transmission [7,64].

## Competing interests

The author(s) declare that they have no competing interests.

## Authors' contributions

MAR initiated the review and drafted the preliminary manuscript. JSR and PAR provided additional data and contributed to manuscript revisions. All authors read and approved the final manuscript.

## Supplementary Material

Additional File 2 Sequence and alignment of World Health Organisation designated measles virus reference strains. Aligned sequence of the 456 nucleotides at the COOH terminus of the N protein for each measles virus reference strain as designated by the World Health Organisation. These sequences should be used in phylogenetic analysis to determine measles virus genotype of newly derived sequence.
Click here for file

Additional File 1 Table: Temporal and geographical distribution of measles of measles virus genotypes 1950 – 2004 (data reflects publications available as of August 2005). Measles virus genotypes listed alphabetically, by year of circulation, location and associated publication. Click here for file
