# Supplementary material for: Review of the temporal and geographical distribution of measles virus genotypes in the prevaccine and postvaccine eras
Source: Virol J. 2005 Nov 22;2:87. doi: 10.1186/1743-422X-2-87 (PMC1318492; doi:10.1186/1743-422X-2-87)
Supplement: Additional File 2 — Sequence and alignment of World Health Organisation designated measles virus reference strains. Aligned sequence of the 456 nucleotides at the COOH terminus of the N protein for each measles virus reference strain as designated by the World Health Organisation. These sequences should be used in phylogenetic analysis to determine measles virus genotype of newly derived sequence. [file 1743-422X-2-87-S2.doc]

**Additional File 2: Sequence and alignment of World Health Organisation designated Measles Virus reference strains**

**Global Measles Specialised laboratory Centers for Disease Control and Prevention, Atlanta GA USA**

**Contact: Dr Paul Rota email:** [**par1@cdc.gov**](mailto:par1@cdc.gov)

2005N-REF.MSF MSF: 456 TYPE: N MAY 9, 2005 16:07 CHECK: 1331 ..

NAME: MONTREAL.CAN_89_D4 LEN: 456 CHECK: 3809 WEIGHT: 1.00

NAME: NJ.USA_94_D6 LEN: 456 CHECK: 8418 WEIGHT: 1.00

NAME: HUNAN.CHN_93_H1 LEN: 456 CHECK: 8178 WEIGHT: 1.00

NAME: PALAU.BLA_93_D5 LEN: 456 CHECK: 3028 WEIGHT: 1.00

NAME: CHICAGO.USA_89_D3 LEN: 456 CHECK: 107 WEIGHT: 1.00

NAME: ED-WT_A LEN: 456 CHECK: 901 WEIGHT: 1.00

NAME: JM.USA_77_C2 LEN: 456 CHECK: 7706 WEIGHT: 1.00

NAME: BERKELEY.USA_83_G1 LEN: 456 CHECK: 8091 WEIGHT: 1.00

NAME: YAOUNDE.CAE_83_B1 LEN: 456 CHECK: 1325 WEIGHT: 1.00

NAME: LIBREVILLE.GAB_84_B2 LEN: 456 CHECK: 1136 WEIGHT: 1.00

NAME: WTF.DEU_90_C2 LEN: 456 CHECK: 9432 WEIGHT: 1.00

NAME: MADRID.SPA_94_F LEN: 456 CHECK: 151 WEIGHT: 1.00

NAME: MVP.UK_74_D1 LEN: 456 CHECK: 423 WEIGHT: 1.00

NAME: BRAXATOR.DEU_71_E LEN: 456 CHECK: 12 WEIGHT: 1.00

NAME: JOHANN.SOA_88_D2 LEN: 456 CHECK: 697 WEIGHT: 1.00

NAME: BANGKOK.THA_93_D5 LEN: 456 CHECK: 1787 WEIGHT: 1.00

NAME: TOKYO.JPN_84K_C1 LEN: 456 CHECK: 1248 WEIGHT: 1.00

NAME: MANCHES.UNK_94_D8 LEN: 456 CHECK: 1023 WEIGHT: 1.00

NAME: VIC.AUS_85_D7 LEN: 456 CHECK: 844 WEIGHT: 1.00

NAME: NY.USA_94_B3 LEN: 456 CHECK: 68 WEIGHT: 1.00

NAME: IBADAN.NIE_97_B3 LEN: 456 CHECK: 422 WEIGHT: 1.00

NAME: AMSTER.NET_97_G2 LEN: 456 CHECK: 9804 WEIGHT: 1.00

NAME: BEIJING.CHN_94_H2 LEN: 456 CHECK: 9268 WEIGHT: 1.00

NAME: ILLIN.USA_99_D7 LEN: 456 CHECK: 2788 WEIGHT: 1.00

NAME: VIC.AUS_99_D9 LEN: 456 CHECK: 9912 WEIGHT: 1.00

NAME: GRESIK.INO_02_G3 LEN: 456 CHECK: 8245 WEIGHT: 1.00

NAME: UGANDA-01-D10 LEN: 456 CHECK: 2508 WEIGHT: 1.00

//

1 50

MONTREAL.CAN_89_D4 AAGGTCAGTT CCACATTGGC ATCTGAACTC GGTATCACTG CCGAGGATGC

NJ.USA_94_D6 AAGGTCAGTT CCACATTGGC ATCTGAACTC GGTATCACTG CCGAGGATGC

HUNAN.CHN_93_H1 AAGGTCAGTT CCACATTGGC ATCTGAACTC GGTATCACGG CCGAGGATGC

PALAU.BLA_93_D5 AAGGTCAGTT CCACATTGGC ATCTGAACTC GGTATCACTG CCGAGGATGC

CHICAGO.USA_89_D3 AAGGTCAGTT CCACATTGGC ATCCGAACTC GGTATCACTG CCGAGGATGC

ED-WT_A AAGGTCAGTT CCACATTGGC ATCTGAACTC GGTATCACTG CCGAGGATGC

JM.USA_77_C2 AAGGTCAGTT CCACATTGGC ATCTGAACTC GGTATCACTG CCGAAGATGC

BERKELEY.USA_83_G1 AAGGTCAGTT CCACATTGGC ATCTGAACTT GGTATCACGG CCGAGGATGC

YAOUNDE.CAE_83_B1 AAGGTCAGTT CCACATTGGC ATCTGAACTC GGTATCACGG CCGAGGATGC

LIBREVILLE.GAB_84_B2 AAGGTCAGTT CCACATTGGC ATCTGAACTC GGTATCACTG CCGAGGATGC

WTF.DEU_90_C2 AAGGTCAGTT CCACATTGGC ATCTGAACTC GGTATCACTG CCGAAGATGC

MADRID.SPA_94_F AGGGTCAGTT CCACATTGGC ATCTGAACTC GGTATCACTG CCGAGGATGC

MVP.UK_74_D1 AAGGTCAGCT CCACATTGGC ATCTGGACTC GGTATCACTG CCGAGGACGC

BRAXATOR.DEU_71_E AAGGTCAGTT CCACATTGGC ATCTGAACTC GGTATCACTG CCGAGGATGC

JOHANN.SOA_88_D2 AAGGTCAGTT CCACATTAGC ATCTGAACTC GGTATCACTG CCGAGGATGC

BANGKOK.THA_93_D5 AAGGTCAGTT CCACATTGGC ATCTGAACTC GGTATCACTG CCGAGGATGC

TOKYO.JPN_84K_C1 AAGGTCAGTT CCACATTGGC ATCTGAACTC GGTATCACTG CCGAGGATGC

MANCHES.UNK_94_D8 AAGGTCAGTT CCACATTGGC ATCTGAACTC GGTATCACTG CCGAGGATGC

VIC.AUS_85_D7 AAGGTCAGTT CCACATTGGC ATCTGAACTC GGTATCACTG CCGAGGATGC

NY.USA_94_B3 AAGGTCAGTT CCACATTGGC ATCTGAACTC GGTATCACTG CCGAGGATGC

IBADAN.NIE_97_B3 AAGGTCAGTT CCACATTGGC ATCTGAACTC GGTATCACTG CCGAGGATGC

AMSTER.NET_97_G2 AAGGTCAGTT CCACATTGGC ATCTGAACTC GGTATCACGG CCGAGGATGC

BEIJING.CHN_94_H2 AAGGTCAGTT CCACATTAGC ATCTGAACTC GGTATCACGG CCGAGGATGC

ILLIN.USA_99_D7 AAGGTCAGTT CTACATTGGC ATCTGAACTC GGTATCACTG CCGAGGATGC

VIC.AUS_99_D9 AAGGTCAGTT CCACATTGGC ATCTGAACTC GGTATCACTG CCGAGGATGC

GRESIK.INO_02_G3 AAGGTAAGTT CCACATTGGC ATCTGAACTC GGTATCACAG CCGAGGATGC

UGANDA-01-D10 AAGGTCAGTT CCACATTAGC ATCTGAACTC GGTATCACTG CCGAGGATGC

51 100

MONTREAL.CAN_89_D4 AAGGCTTGTT TCAGAGATTG CAATGCATAC TACTGAGGAC AGGATCAGTA

NJ.USA_94_D6 AAGGCTTGTC TCAGAGATTG CAATGCATAC TACTGAGGAC AGGATCAGTA

HUNAN.CHN_93_H1 AAGGCTTGTT TCAGAGATTG CAATGCATAC TACTGAGGAC AGGACCAGTA

PALAU.BLA_93_D5 GAGGCTTGTT TCAGAGATTG CAATGCATAC TACTGAGGAC AGGATCAGTA

CHICAGO.USA_89_D3 AAGGCTTGTT TCAGAGATTG CAATGCATAC TACTGAGGAC AGGATCAGTA

ED-WT_A AAGGCTTGTT TCAGAGATTG CAATGCATAC TACTGAGGAC AAGATCAGTA

JM.USA_77_C2 AAGGCTTGTT TCAGAGATCG CAATGCATAC TACAGAGGAC AGGATCAGTA

BERKELEY.USA_83_G1 GAGGCTTGTT TCAGAGATTG CAATGCATAC CACTGAGGAC AGGATCAGCA

YAOUNDE.CAE_83_B1 AAGGCTTGTT TCAGAGATTG CAATGCATAC TGCTGAGGAC AGGATCAGTA

LIBREVILLE.GAB_84_B2 AAGGCTTGTT TCAGAGATTG CAATGCATAC TACTGAGGAC AGGATCAGTA

WTF.DEU_90_C2 AAGGCTTGTT TCAGAGATCG CAATGCATAC TACAGAGGAC AGGATCAGTA

MADRID.SPA_94_F AAGACTTGTT TCAGAGATTG CGATGCATAC TACTGAGGAC AGGATCAGTA

MVP.UK_74_D1 AAGGCTTGTT TCAGAGATTG CAATGCATAC TACTGAGGAC AGGATCAGTA

BRAXATOR.DEU_71_E AAGGCTTGTT TCGGAGATTG CAATGCACAC TACTGAGGAC AGGATCAGCA

JOHANN.SOA_88_D2 AAGGCTTGTT TCAGAGATTG CAATGCATAC TACTGAGGAC AGGATCAGTA

BANGKOK.THA_93_D5 GAGGCTTGTG TCAGAGATTG CAATGCATAC TACTGAGGAC AGGATCAGTA

TOKYO.JPN_84K_C1 AAGGCTTGTT TCAGAGATTG CAATGCACAC TACTGAGGAC AGGATCAGTA

MANCHES.UNK_94_D8 AAGGCTTGTT TCAGAGATTG CAATGCATAC TACTGAGGAC AGGACCAGTA

VIC.AUS_85_D7 AAGGCTTGTT TCAGAGATCG CAATGCATAC TACTGAGGAC AGGACCAGTA

NY.USA_94_B3 AAGGCTTGTT TCAGAGATTG CAATGCATAC TACTGAGGAC AGGATCAGTA

IBADAN.NIE_97_B3 AAGGCTTGTT TCAGAGATTG CAATGCATAC TACTGAGGAC AGGATCAGTA

AMSTER.NET_97_G2 AAGGCTTGTT TCAGAGATTG CAATGCATAC TACTGAGGAC AGGATCAGTA

BEIJING.CHN_94_H2 AAGGCTTGTT TCAGAAATTG CAATGCATAC TACTGAAGAC AGGATCAGCA

ILLIN.USA_99_D7 AAGGCTTGTT TCAGAAATCG CAATGCATAC TACTGAGGAC AGGACCAGTA

VIC.AUS_99_D9 AAGACTTGTT TCAGAGATTG CAATGCATAC TACTGAGGAC AGGATCAGTA

GRESIK.INO_02_G3 AAGGCTTGTT TCAGAGATTG CAATGCATAC TACTGAGGAC AGGACCAGTA

UGANDA-01-D10 AAGGCTTGTT TCAGAGATTG CAATGCATAC TACTGAGGAC AGGATCAGTA

101 150

MONTREAL.CAN_89_D4 GAGCGGTTGG ACCCAGACAA GCCCAAGTGT CATTTATACA CGGTGATCAA

NJ.USA_94_D6 GAGCGGTCGG ACCCAGACAA GCCCAAGTGT CATTTCTACA CGGTGATCAA

HUNAN.CHN_93_H1 GAGCGGTTGG ACCCAGGCAA GCCCAAGTCT CATTTTTACA CGGTGATCAA

PALAU.BLA_93_D5 GAGCGGTCGG ACCCAGACAA GCCCAAGTGT CATTTCTACA CGGTGATCAA

CHICAGO.USA_89_D3 GAGCGGTCGG ACCCAGACAA GCCCAAGTAT CATTTCTACA CGGTGATCAA

ED-WT_A GAGCGGTTGG ACCCAGACAA GCCCAAGTAT CATTTCTACA CGGTGATCAA

JM.USA_77_C2 GAGCGGTTGG ACCCAGACAA TCCCAAGTGT CATTCCTACA CGGTGATCAA

BERKELEY.USA_83_G1 GAGCGGTTGG ACCCAGGCAG GCCCAAGTGT CATTTCTACA CGGTGATCAA

YAOUNDE.CAE_83_B1 GAGCAGTTGG ACCTAGACAA GCCCAAGTGT CATTCCTACA CGGTGATCAA

LIBREVILLE.GAB_84_B2 GAGCGGTTGG GCCCAGACAA GCCCAAGTAT CATTTCTACA CGGTGATCAA

WTF.DEU_90_C2 GAGCGGTTGG ACCCAGACAA TCCCAAGTGT CATTCCTACA CGGTGATCAA

MADRID.SPA_94_F GAGCGGTTGG ACCCAGACAA GCCCAAGTGT CATTTCTACA CGGTGATCAA

MVP.UK_74_D1 GAGCGGTTGG ACCCAGACAA GCCCAAGTGT CATTTCTACA CGGTGATCAA

BRAXATOR.DEU_71_E GAGCGGTTGG ACCCAGACAA GCCCAAGTGT CATTTCTACA CGGTGATCAA

JOHANN.SOA_88_D2 GAGCGGTTGG ACCCAGACAA GCCCAAGTAT CATTTCTACA CGGTGATCAA

BANGKOK.THA_93_D5 GAGCGGTCGG ACCCAGACAA GCCCAAGTGT CATTTCTACA CGGTGATCAA

TOKYO.JPN_84K_C1 GAGCGGTTGG ACCCAGACAA GCCCAAGTGT CATTTCTACA CGGTGATCAA

MANCHES.UNK_94_D8 GAGCAGTTGG ACCCAGACAA GCTCAAGTGT CATTTCTACA CGGTGATCAA

VIC.AUS_85_D7 GAGCGGTTGG ACCCAGACAA GCCCAAGTGT CATTTCTACA CGGTGATCAA

NY.USA_94_B3 GAGCAGTTGG ACCCAGACAA GCCCAAGTGT CATTCCTACA CGGTGATCAA

IBADAN.NIE_97_B3 GAGCAGTTGG ACCCAGACAA GCCCAAGTGT CATTTCTACA CGGTGATCAA

AMSTER.NET_97_G2 GAGCGGTTGG ACCCAGGCAA GCCCAAGTGT CATTTCTACA CGGTGATCAA

BEIJING.CHN_94_H2 GAGCGGTTGG ACCCAGGCAA GCCCAAGTGT CATTTCTACA CGGTGATCAA

ILLIN.USA_99_D7 GAGCGGTTGG ACCCAGACAG GCCCAAGTGT CATTTCTGCA CGGTGATCAA

VIC.AUS_99_D9 GAGCAGTCGG ACCCAGACAA GCCCAAGTGT CATTTCTACA CGGTGATCAA

GRESIK.INO_02_G3 GAGCGGTTGG ACCCAGGCAA GCTCAAGTGT CATTTCTACA CGGTGATCAA

UGANDA-01-D10 GAGCGGTTGG ACCCAGACAA GCCCAGGTGT CATTTCTACA CGGTGATCAA

151 200

MONTREAL.CAN_89_D4 AGTGAGAATG AGCTACCAGG ATTGGGGGGC AAGGAAGATA GGAGGGTCAA

NJ.USA_94_D6 AGTGAGAATG AGCTACCAGG ATTGGGGGGC AAGGAAGATA GGAGAGCCAA

HUNAN.CHN_93_H1 AGTGAGAATG AGCTACCGGG ATTGGGGAGC AAGGAAGATA GAAGGGTCAA

PALAU.BLA_93_D5 AGTGAGAATG AGCTCCCAGG ATTGGGGGGC AAGGAAGATA GGAGGGTCAA

CHICAGO.USA_89_D3 AGTGAGAATG AGCTACCAGG ATTGGGGGGC AAGGAAGACA GGAGGGTCAA

ED-WT_A AGTGAGAATG AGCTACCGAG ATTGGGGGGC AAGGAAGATA GGAGGGTCAA

JM.USA_77_C2 AATGAAAATG AGCTACCGAG ATGGGGGGGT AAGGAAGATA TGAGGGTCAA

BERKELEY.USA_83_G1 AGTGAGAACG AGCTACCGGG ATTGGGGGGG AAGGAAGACA GGAGGGTCAA

YAOUNDE.CAE_83_B1 AGTGAGAATG AGCTGCCGAG ATTGGGGGGC AAGGAGGACA GGAGGGTCAA

LIBREVILLE.GAB_84_B2 AGTGAGAATG AGCTGCCGAG ATTGGGGGGC AAGGAGGATA GAAGGGTCAA

WTF.DEU_90_C2 AATGAAAGTG AGCTACCGAG ATGGGGGGGT AAGGAAGATA TGAGGGTCAA

MADRID.SPA_94_F AGTGAGAATG AGCTACCGAG ATTGGGGGGC AAGGAAGACA GGAGGATCAA

MVP.UK_74_D1 AGTGAGAATG AGCTACCAGG ATTGGGGGGC AAGGAAGACA GGAGGGTCAA

BRAXATOR.DEU_71_E AGTGAGAATG AGCTGCCGAG ATGGGGGGGC AAGGAAGATA GGAGGGCCAA

JOHANN.SOA_88_D2 AGTGAGAATG AGCTACCAGG ATTGGGGGGC AAGGAAGATA GGAGGGTCAA

BANGKOK.THA_93_D5 AGTGAGAATG AGCTACCAGG ATTGGGGGGC AAGGAAGATA GGAGGGTCAA

TOKYO.JPN_84K_C1 AGTGAAAATG AGCTACCGAG ATGGGGGGGC AAGGAAGATA TGAGGGTCAA

MANCHES.UNK_94_D8 AGTGAGAATG AGCTACCAGG ATTGGGGGGC AAGGAAGATA GGAGGGTCAG

VIC.AUS_85_D7 AGTGAGAATG AGCTACCAGG ATTGGGGGGC AAGGAAGACA GGAGGGTCAA

NY.USA_94_B3 AGTGAGAATG AGCTGCCGAG ATTGGGGGGC AAGGAGGACA GGAGGGTCAA

IBADAN.NIE_97_B3 AGTGAGAATG AGCTGCCGAG ATTGGGGGGC AAGGAGGACA GGAGGGTCAA

AMSTER.NET_97_G2 AGTGAGAATG AGCTACCGGG ATTGGGAGGT AAGGAAGATA AGAGAGTCAA

BEIJING.CHN_94_H2 AGTGAGAACG AGCTACCGGG ATTAGGGGTC AAGGAAGATA GGAGGGTCAA

ILLIN.USA_99_D7 AGTGGGAGTG AGCTACCAGG ATTGGGGGGT AGGGAGGACA GGAGGGTCGG

VIC.AUS_99_D9 AGTGAGAATG AGCTACCAGG ACTGGGGGGC AAGGAAGATA GGAGGGTCAA

GRESIK.INO_02_G3 AGTGAGAATG AGCTACCGGG ATTGGGGGGT AAGGAAGATA AGAAGGTCAA

UGANDA-01-D10 AGTGAGAATG AGCTACCAGG ATTGGGAGGC AAGGAAGATA GGAGGGTCAA

201 250

MONTREAL.CAN_89_D4 ACAGAGTCGG GGAGAAGCCA GGGAGAGCTA CAGAGAAACC GGGTCCAGTA

NJ.USA_94_D6 ACAGAGCCGA GGAGAAGCCA GGGATAGCTA CAGAGAAACT GGGTCCAGCA

HUNAN.CHN_93_H1 ACAGAGTCGA GGGGAAACCA GGGAGAACTC CAGAGAAACC GGGCCCAGCA

PALAU.BLA_93_D5 ACAGAGTCGG GGAGAAGCCA GGGAGAGCTA CAGAGAGACC GGGTCCAGCA

CHICAGO.USA_89_D3 ACAGAGTCGG GGAGAAGCCA GGGAGAGCTA CAGAGAAACC GGGTCCAGCA

ED-WT_A ACAGAGTCGA GGAGAAGCCA GGGAGAGCTA CAGAGAAACC GGGCCCAGCA

JM.USA_77_C2 ACAGAGTCGG GGAGAAGCCA GAGAGAGCTA CAGAGAAACC AGGCCCAGCA

BERKELEY.USA_83_G1 ACAGAGTCGA GGAGAAACCA GGGAGAGCTA CAGAGAAACC GGGCCCAGCA

YAOUNDE.CAE_83_B1 ACAGAGTCGA GGAGAAGCCG GGGAGAGCTA CAGAGAAACC GGGCCCAGCA

LIBREVILLE.GAB_84_B2 ACAGAGCCGA GGAGAAGCCG GGGAGAGCTA CAGAGAAACT GGGCCCAGCA

WTF.DEU_90_C2 ACAGAGTCGG GGAGAAGCCA GAGAGAGTTA CAGAGAAACC GGGCCCAGCA

MADRID.SPA_94_F ACAGAGTCAA GGAGAAGCCA GGGAAAGCTA CAGAGAAACC GGGCCCAGCA

MVP.UK_74_D1 ACAGAGTCGA GGAGAAGCCA GGGAGAGCTA CAGAGATACC GGGTCCAGCA

BRAXATOR.DEU_71_E ACAGAGTCGA GGAGAAGCCA GGGAGATCTA CAGAGAAACC GGGCCCAGCA

JOHANN.SOA_88_D2 ACAGAGTCGG GGAGAAGCCA GGGAGAGCTA CAGAGAAACC GGGTCCAGCA

BANGKOK.THA_93_D5 ACAGAGTCGG GGAGAAGCCA GGGAGAGCTA CAGAGAAACC GGGTCCAGCA

TOKYO.JPN_84K_C1 ACAGAGTCGA GGAGAAGCCA GGGAGAGCTA CAGAGAAACC GGGCCCAGTA

MANCHES.UNK_94_D8 ACAGAGTCGG GGAGAAGCCA GGGAGAGCAA CAGAGAAACC GGGTCCAGCA

VIC.AUS_85_D7 ACAGGGTCGG GGAGAAGCCA GGGAGAGCTA CAGAGAAACC AGGTCCAGCA

NY.USA_94_B3 ACAGGGTCGA GGAGAAGCCG GGGAGAGCTA CAGAGAAACC GGGCCCAGCA

IBADAN.NIE_97_B3 ACAGAGCCGA GGAGAAGCCG GGGAGAGCCA CAGAGAAACC GGGCCCAGCA

AMSTER.NET_97_G2 ACAGAGTCGA GGAGAAGCCA GGGAGAGCTA TAGAGAAACT GGGCACAGCA

BEIJING.CHN_94_H2 ACAGAGTCGA GGAGAAGCCA GGGAGAGCCC CAGAGAAACC GGGCCCAACA

ILLIN.USA_99_D7 GCAGAGTCGG GGAGAAGCCA GGGAGAGCTA CAGAGAGACC GGGTCCAGCA

VIC.AUS_99_D9 ACAGAGTCGG GGAGAAGCAA GGGAGAGCTA CAGAGAAGCC GGATCCAGCA

GRESIK.INO_02_G3 ACAGAGTCGA GGAGAAGCCA GGGAGAGCTA TAGAGAAACC GGGCCCAGCA

UGANDA-01-D10 ACAGAGTCGA GGAGAAGCCA TGGAGAGCCA CAGAGAAACC GGGTCCAGCA

251 300

MONTREAL.CAN_89_D4 GAGCAAGTGA TGCGAGAGCT GCCCATCTTC CAACCAGCAC ACCCCTAGAC

NJ.USA_94_D6 GAGCAAGTGA TGCAAGAGCT GCCCATCTTC CAACCAGCAC ACCCCTAGAC

HUNAN.CHN_93_H1 GATCAAGTGA TGCGAGAGCT GCCCATCTCC CAACCAGCAC ACCCCCAGAC

PALAU.BLA_93_D5 GAGCAAGTGA TGAGAGAGCT GCCCATCTTC CAACCAGCAC ACCCCTAGAC

CHICAGO.USA_89_D3 GAGCAAGTGA TGCGAGAGCT GCCCATCCTC CAACCAGCAT GCCCCTAGAC

ED-WT_A GAGCAAGTGA TGCGAGAGCT GCCCATCTTC CAACCGGCAC ACCCCTAGAC

JM.USA_77_C2 GAGCAAGTGA CGCGAGAGCT ACCCATCCTC CAACCGACAC ACCCTTAGAC

BERKELEY.USA_83_G1 GAGCAGGTGA TGCGAGAGCT GCCCACCTTC CAACCGGCAC ACTCCTAGAC

YAOUNDE.CAE_83_B1 GAGCAAGTGA TGCAAGAGCT GCCCATCCTC CGACCGGCAC ACCCCTAGAC

LIBREVILLE.GAB_84_B2 GAGCAAGTGA TGCGAGAGCC ACCCATCTTC CAACCGGCAC ACCCCTAGAC

WTF.DEU_90_C2 GAGCAAGTGA CGCGAGAGCT GCCCATCCTC CAACCGACAC ACCCTTAGAC

MADRID.SPA_94_F GAGCAAGTGA CGCGAGGGCT GCCCATCTTC CAACCGGCAC ACCCCTAGAT

MVP.UK_74_D1 GAGCAAGTGA TGCAAGAGCT GCCCATCTTC CAACCAGCAC ACCCCTAGAC

BRAXATOR.DEU_71_E GAGCAAGTGA TGCGAGAGCT GCCCATCTTC CAACCGGCAC ACCCCTAGAC

JOHANN.SOA_88_D2 GAACAAGCGA TGCGAGAGCT GCCCATCTTC CAACCAGCAC ACCCCTAGAC

BANGKOK.THA_93_D5 GAGCAAGTGA TGCGAGAGCT GCCCATCTTC CAACCGGCAC ACCCCTAGAC

TOKYO.JPN_84K_C1 GAGCAAGTGA TGCGAGAGCT GCCCATCTTC CAACCGACAC ACCCCTAGAC

MANCHES.UNK_94_D8 GATTAAGTGA TGCGAGAGCT GCCCATCTTC CAACCAGCAC ACCCCTAGAC

VIC.AUS_85_D7 GAGCAAGTGA TGCGAGAGCT GCCCATCTTC CAACCAGCAC ACCCCAAGAC

NY.USA_94_B3 GAGCAAGTGA TGCGAGAGCT GCCCATCCTC CAACCGGCAC ACCCCTAGAC

IBADAN.NIE_97_B3 GAGCAAGTGA TGCGAGAGCT GCCCATCCTC CAACCGGCAC ACCCTTAGAC

AMSTER.NET_97_G2 GAGCAAATGA TGCGAGAGCT GCTGACCTTC CAACCGGCAC ACCCCTAGAC

BEIJING.CHN_94_H2 GAACAAGTGA TGTGAGAGTT GCCCATCTCC CAACCAGCAC ACCCCCAGAC

ILLIN.USA_99_D7 GAGCAAGTGA TGCGAGAGCT GCCCATCTTC CAACCAGCAC ACCCCTAGAC

VIC.AUS_99_D9 GAGCAGGTGA TGAGAGAGCT GCCCATCTTC CAACCAGCAC ACCCCTAGAC

GRESIK.INO_02_G3 GAGCAAATGA TGCGAGAGCT GCCCACCTTC CAACCGGCAC ACCCCTAGAC

UGANDA-01-D10 GAACAAGTGA TGTGAGAGCT GTCCATCTTC CAACCAGCAC ACCCCTAGAC

301 350

MONTREAL.CAN_89_D4 ATTGACACTG CATCAGAGTC AGGCCAAGAT CCGCAGGACA GTCGAAGGTC

NJ.USA_94_D6 ATTGACACTG CATCGGAGAC AAGCCAAGAT CTGCAAGACA GTCGAAGGTC

HUNAN.CHN_93_H1 ATTGACACTG CATCGGAGTA CAGCCAAGAC CCACAGGACA GTCGAAGGTC

PALAU.BLA_93_D5 ATTGACACTG CATCGGAGTC AGGCCAAGAT CCGCAGGACA GTCGAAGGTC

CHICAGO.USA_89_D3 ATTGACACTG CATCGGAGTC AGGCCAAGAT CCGCAGGACA GTCGAAGGTC

ED-WT_A ATTGACACTG CATCGGAGTC CAGCCAAGAT CCGCAGGACA GTCGAAGGTC

JM.USA_77_C2 ATTGACACTG CATCGGAGTC CAGCCAAGAT CCGCAGGACA GTCGAAGGTC

BERKELEY.USA_83_G1 ATTGACACTG CATCGGAGTC CAGCCAAGAC CCACAGGACA GTCGAAGGTC

YAOUNDE.CAE_83_B1 ATTGACACTG CATCGGAGTT CAGCCAAGAT CCGCAGGACA GTCGAAGGTC

LIBREVILLE.GAB_84_B2 ATTGACACTG TATCGGAGTC CAGCCTAGAT CCGCAGGACA GTCGAAGGTC

WTF.DEU_90_C2 ATTGACACTG CATCGGAGTC TAGCCAAGAT CCGCAGGACA GTCGAAGGTC

MADRID.SPA_94_F ATTGACACTG CATCAGAGTC CAGCCAAGAT CCGCTGGACA GTCGAAGGTC

MVP.UK_74_D1 ATTGACACTG CATCGGAGTC AAGCCAAGAT CCTCAGGACA GTCGAAGGTC

BRAXATOR.DEU_71_E ATTGACACTG CATCGGAGTC CAGCCAAGAT CAGCAGGACA GTCAAAGGTC

JOHANN.SOA_88_D2 ATTGACACTG CATCGGAGTC AAGCCAAGAT CCGCAGGACA GTCGAAGGTC

BANGKOK.THA_93_D5 ATTGACACTG CATCGGAGTC AGGCCAAGAT CCGCAGGACA GTCGAAGGTC

TOKYO.JPN_84K_C1 ATTGACACTG CATCGGAGTT CAGCCAAGAT CCGCAGGACA GTCGAAGGTC

MANCHES.UNK_94_D8 ATTGACACTG CATCGGAGTC AGGCCAAGAT CCGCAGGACA GTCGAAGGTC

VIC.AUS_85_D7 ATTGACACTG CATCGGAGTC AGGCCAAGAT CCGCAGGACA GCCGACGGTC

NY.USA_94_B3 ATTGACACTG CATCGGAGTT CAGCCAAGAT CCGCAGGACA GTCGAAGGTC

IBADAN.NIE_97_B3 ATTGACACTG CATCGGAGTT CAGCCAAGAT CCGCAGGACA GTCGAAGGTC

AMSTER.NET_97_G2 ATTGACACTG CATCGGAGTT CAGCCAAGAC CCACAGGACA GTCGAAGGTC

BEIJING.CHN_94_H2 ATTGACACTG CATCGGAGTA CAGCCAAGAC CCACAGGACA GTCGAAGGTC

ILLIN.USA_99_D7 ATTGACACTG CATCGGAGTC AGGCCAAGAT CTGCAGGACA GCCGACGGTC

VIC.AUS_99_D9 ATTGACACTG CATCAGAGTC AGGCCAAGAC CCGCAGGACA GTCGAAGGTC

GRESIK.INO_02_G3 ATTGACACTG CATCGGAGTT CAGCCAAGAC CCACAAGACA GTCGAAGGTC

UGANDA-01-D10 ATTGACACTG CATCGGAGTC AAGTCGAGAT CCGCAGGACA GTCGAAGGTC

351 400

MONTREAL.CAN_89_D4 AGCTGACGCC TTGCTCAGGT TGCAGGCCAT GGCAGGAATC TTGGAAGAAC

NJ.USA_94_D6 AGCTGACGCC CTGCTCAGGC TGCAAGCCAT GGCAGGAATC TCGGAAGAGC

HUNAN.CHN_93_H1 AGCTGACGCC CTGCTCAGGC TGCAAGCCAT GGCAGGGATC CTGGAAGAAC

PALAU.BLA_93_D5 AGCTGACGCC CTGCTCAGGC TGCAAGCCAT GGCAGGAATC TTGGAGGAAC

CHICAGO.USA_89_D3 AGCTGACGCT CTGCTCAGGC TGCAAGCCAT GGCAGGAATC TTGGAAGAAC

ED-WT_A AGCTGACGCC CTGCTTAGGC TGCAAGCCAT GGCAGGAATC TCGGAAGAAC

JM.USA_77_C2 AGCTGACGCC CTGCTCAGGC TGCAAGCCAT GGCAGGAATC TCGGAAGAAC

BERKELEY.USA_83_G1 GGCTGACGCC CTGCTCAGGC TGCAAGCCAT GGCAGGAATC TCGGAAGAAC

YAOUNDE.CAE_83_B1 AGCTGACGCC CTGCTTAGGC TGCAAGCCAT GGCAGGAATC TCGGAAGAAC

LIBREVILLE.GAB_84_B2 AGCTGACGCC CTGCTTAGGC TGCAAGCCAT GGCAGGAATC TCGGAAGAAC

WTF.DEU_90_C2 ACGTGACGCT CTGCTCAGGC TGCAAGCCAT GGCAGGAATC TCGGAAGAAC

MADRID.SPA_94_F AGCTGAAGCC CTGCTCAGGC TGCAAGCCAT GGCAGGAATC TCGGAAGAAC

MVP.UK_74_D1 AGCTGACGCC CTGCTCAGGC TGCAAGCCAT GGCAGGAATC TCGGAAGAAC

BRAXATOR.DEU_71_E AGCTGACGCC CTACTCAGGC TGCAAGCCAT GGCAGGAATC TCGGAAGAAC

JOHANN.SOA_88_D2 AGCTGACGCC CTGCTTAGGC TGCAAGCCAT GGCAGGAATC TCGGAAGAAC

BANGKOK.THA_93_D5 AGCTGACGCC CTGCTCAGGC TGCAAGCCAT GGCAGGAATC TTGGAAGAAC

TOKYO.JPN_84K_C1 AGCTGAAGCC CTGCTCAGGC TGCAAGCCAT GGCAGGAATC TCGGAAGAAC

MANCHES.UNK_94_D8 AGCTGACGCC CTGCTCAGGC TGCAAGCCAT GGCAGGAATC CTGGAAGAAC

VIC.AUS_85_D7 AGCTGACGCC CTGCTCAGGC TGCAAGCCAT GGCAGGAATC TTGGAAGAAC

NY.USA_94_B3 AGCCGACGCC CTGCTTAGGC TGCAAGCCAT GGCAGGAATC TCGGAAGAAC

IBADAN.NIE_97_B3 AGCCGATGCC CTGCTTAGGC TGCAAGCCAT GGCAGGAATC TCGGAAGAAC

AMSTER.NET_97_G2 AGCTGACGCC CTGCTCAGGC TGCAAGCCAT GGCAGGGATC CCGGAAGAAC

BEIJING.CHN_94_H2 AGCTGACGCC TTGCTCAGGC TGCAAGCCAT GGCAGGGATT TTGGAAGAAC

ILLIN.USA_99_D7 AGCTGACGCC CTGCTCAGGC TGCAAGCCAT GGCAGGAATC TTGGAAGAAC

VIC.AUS_99_D9 AGCTGACGCC CTGCTCAGGC TGCAAGCCAT GGCAGGAATC TTGGAAGAAC

GRESIK.INO_02_G3 AGCTGACGCC CTGCTCAGGC TGCAAGCCAT GGCAGGAATC TCGGAAGAAC

UGANDA-01-D10 AGCTGAAGCC CTACTCAGGC TGCAAGCCAT GGCAGGAATC TCGGAAGAAC

401 450

MONTREAL.CAN_89_D4 AAGGCTCAGA TACGGACATC CCTAGGGTGT ACAATGACAA AGATCTTCTA

NJ.USA_94_D6 AAGGCTCAGA CACGGACACC CCCAGAGTGT ACAATGACAG AGATCTTCCA

HUNAN.CHN_93_H1 AAGGCTCAGA CACGGACACC CCTAGAGTGT ACAACGATAG AGATCTTCTA

PALAU.BLA_93_D5 AAGGCTCAGA CACGGACACC CCTAGGGTGT ACAATGACAG TGATCTTCTA

CHICAGO.USA_89_D3 AAGGCTCAGA CACGGACACC CCTAGGGTAT ACAATGACAG AGATCTTCTA

ED-WT_A AAGGCTCAGA CACGGACACC CCTATAGTGT ACAATGACAG AAATCTTCTA

JM.USA_77_C2 AAGGCTCAGA CACGGACACC CCTAGAGTGT ACAATGACAG AGATCTTCTA

BERKELEY.USA_83_G1 AAGGCTCAGA CATGGACACC CTTAGAGTGT ACAATGACAG AGATCTTCTA

YAOUNDE.CAE_83_B1 AAGGCTCAGA CACGGACACC CCTAGAGTGT ACAATGACAG AGATCTTCTA

LIBREVILLE.GAB_84_B2 AAGGCTCAGA CACGGACACC CCTAGAGTGT ACAATGACAG AGATCTGCTA

WTF.DEU_90_C2 AAGGCTCAGA CACGGACACC CCTAGAGTGT ACAATGACAG AGACCTTCTA

MADRID.SPA_94_F AAGGCTCAGA CACGGACACC CCTAGAGTGT ACAATGACAG AGATCTTCTA

MVP.UK_74_D1 AAGGCTCAGA CACGGACACC CCTCGAGTGT ACAATGACAG AGATCTTCTA

BRAXATOR.DEU_71_E AAGGCTCAGA CACGGACACC CCTAGAGTGT ACAATGACAG AGATCTTCTA

JOHANN.SOA_88_D2 AAGGCTCAGA CACGGACACC CCTAGAGTGT ACAATGACAG AGATCTTCTA

BANGKOK.THA_93_D5 AAGGCTCAGA CACGGACACC CCTAGGGTGT ACAATGACAG AGATCTTCTA

TOKYO.JPN_84K_C1 AAGGCTCAGA CACGGACACC CCTAGAGTGT ACAATGACAG AGATCTTCTA

MANCHES.UNK_94_D8 AAGGCTCAGA CACGGACACC CCCAGGGTGT ACAATGACAG AAATCTTCTA

VIC.AUS_85_D7 AAGGCTCAGA CACGGACACC CCTAGGGTGT ACAATGATAG AGATCTTCTA

NY.USA_94_B3 AAGGCTCAGA CACGGACACC CCTAGAGTGT ACAATGGCAG AGACCTTCTA

IBADAN.NIE_97_B3 AAGACTCAGA CACGGACACC CCTAGAGTGT ACAATGATAG AGACCTTCTA

AMSTER.NET_97_G2 AAGGCTCAGA CATGGACACC CCTAGAGTGT ACAATGACAG AGATCTTCTA

BEIJING.CHN_94_H2 AAAGCTCAGA CACGGACACC CCTAGAGTGT ACAACGACAG AGATCTTCTA

ILLIN.USA_99_D7 AAGGCTCAGA CACGGACACC CCTAGGGTGT ACAATGACAG AGATCTTCTA

VIC.AUS_99_D9 AAGGCTCAGA CACGGACACC CCTAGGGTGT ACAATGACAG AGATCTTCTA

GRESIK.INO_02_G3 AAGGCTCAGA CATGGACACC CCTAGAGTGT ACAATGACAG AGATCTTCTA

UGANDA-01-D10 AAGGCTCAGA CACGGACACC CCTAGAGTGT ACAATGACAG AGATCTACTA

451

MONTREAL.CAN_89_D4 GACTAG

NJ.USA_94_D6 GACTAG

HUNAN.CHN_93_H1 GACTAG

PALAU.BLA_93_D5 GACTAG

CHICAGO.USA_89_D3 GACTAG

ED-WT_A GACTAG

JM.USA_77_C2 GACTAG

BERKELEY.USA_83_G1 GACTAG

YAOUNDE.CAE_83_B1 GACTAG

LIBREVILLE.GAB_84_B2 GACTAG

WTF.DEU_90_C2 GACTAG

MADRID.SPA_94_F GACTAG

MVP.UK_74_D1 GACTAG

BRAXATOR.DEU_71_E GACTAG

JOHANN.SOA_88_D2 GACTAG

BANGKOK.THA_93_D5 GACTAG

TOKYO.JPN_84K_C1 GACTAG

MANCHES.UNK_94_D8 GACTAG

VIC.AUS_85_D7 GACTAG

NY.USA_94_B3 GACTAG

IBADAN.NIE_97_B3 GACTAG

AMSTER.NET_97_G2 GACTAG

BEIJING.CHN_94_H2 GACTAG

ILLIN.USA_99_D7 GACTAG

VIC.AUS_99_D9 GACTAG

GRESIK.INO_02_G3 GACTAG

UGANDA-01-D10 GACTAG
