# Supplementary material for: Review of the temporal and geographical distribution of measles virus genotypes in the prevaccine and postvaccine eras
Source: Virol J. 2005 Nov 22;2:87. doi: 10.1186/1743-422X-2-87 (PMC1318492; doi:10.1186/1743-422X-2-87)
Supplement: Additional File 1 — Table: Temporal and geographical distribution of measles of measles virus genotypes 1950 – 2004 (data reflects publications available as of August 2005). Measles virus genotypes listed alphabetically, by year of circulation, location and associated publication. [file 1743-422X-2-87-S1.doc]

Additional File 1:

Review of the temporal and geographical distribution of measles virus genotypes in the prevaccine and postvaccine eras.

Michaela A Riddell^1*^, Jennifer S Rota^2^ and Paul A Rota^2^

^1^ Scientist/PhD Scholar, Victorian Infectious Diseases Reference Laboratory/WHO Western Pacific Measles Regional Reference Laboratory and Department of Public Health, School of Population Health, University of Melbourne, Parkville 3010, Victoria, Australia.

^2^ Centers for Disease Control and Prevention, Atlanta, GA, 30333 USA

*Current address: Dept. Molecular Microbiology and Immunology, Johns Hopkins School of Public Health, Baltimore, MD, 21205 USA

Email addresses: Michaela Riddell: [michaela.riddell@mh.org.au](mailto:michaela.riddell@mh.org.au)

Jennifer Rota: [jjs4@CDC.GOV](mailto:jjs4@CDC.GOV)

Paul Rota: [par1@cdc.gov](mailto:par1@cdc.gov)

Temporal and geographical distribution of measles virus genotypes 1950 – 2004

(data reflects publications available as of August 2005)

**(*country)-** country specified confirmed as source of importation/history of travel to country specified within accepted incubation dates for disease

**(?country)** – history of recent travel to country specified, however not confirmed as source of importation

**(*unknown)** –source country of importation not known

** Unique type strain, sequence analyses revealed isolate least divergent from wild-type A strain [1]

^#^ Indicates suspected year of original infection for SSPE cases

Personal communication Doris Chibo, VIDRL, Melbourne Australia

| Genotype | Year of circulation | Country where measles identified (*country identified as source of importation) | Published reference |
| --- | --- | --- | --- |
| A  (Wild-type) | 1950s - 1960s  1956^#^  1960  1970s  1988; 1996  1990 – 1998 | Japan & Russia & Finland & Romania & USA  Northern Ireland (SSPE case**)  Czech Rep. & Slovakia  Brazil  Russia  USA & UK (*Russia) & Russia & China & Argentina | [2, 3]  [1]  [4]  [5]  [4]  [2, 6-10] |
| B1 | 1983 | Cameroon | [3, 11] |
| B2 | 1984  2002; 2003 | Gabon  South Africa (*Angola); Angola | [3, 11]  [12] |
| B3 | 1993  1994  1994; 1995  1994 – 1998  1997 – 1998  1998; 2001; 2002  1997 – 2003  2000  2001, 2003  2001  2001  2002  2002: 2003 | Gambia  France (*West Africa)  USA (*Kenya)  Gambia & Nigeria  Nigeria & Ghana  UK (*unknown; unknown; unknown)  Sudan  Germany (*unknown)  Spain (*Algeria, Equatorial Guinea)  Cameroon  Burkino Faso & Democratic Republic of Congo & Republic of Congo  USA (*Nigeria)  Tunisia : Libya | [13]  [14]  [2, 15]  [16]  [17]  [8, 18]  [19, 20]  [21]  [22]  [13]  [23]  [24]  [25] |
| C1 | 1955^#^; 1956^#^  1968^#^; 1971^#^; 1984^#^  Early 1970s  1970 - 1979; 1981  1979  Mid 1980s  1984; 1985  1992; 1993  1991 – 1994 | Northern Ireland (SSPE cases)  Japan (SSPE cases)  USA (SSPE cell line)  Spain (wild-type; SSPE)  Canada  Germany (SSPE cases)  Japan  Germany  Argentina | [1]  [26]  [27]  [22, 27, 28 ]  [29]  [27]  [27, 30]  [27, 28 ]  [31] |
| C2 | 1972 – 1983  1977  Mid 1980s  1984  1990 – 1991  1991; 1992; 1994  1990; 1992; 1993  1992 – 1995  1994; 1995; 1996  1996  1996; 1997; 1998  1996 – 1997  1996; 1997  1997; 1998  1997  1998  1998 – 1999  1999 – 2000; 2002; 2003  2002; 2003 | Denmark  USA  Austria (SSPE)  Canada  Australia  Netherlands  Czech Rep. & Slovakia & Spain; Germany  UK  USA (*unknown; Italy; Germany)  Brazil  UK (*Europe)  Luxembourg (*Belgium & unknown)  Germany; Denmark  USA (*Germany; Zimbabwe)  Canada (*Germany & France)  Netherlands  Morocco  Germany  Spain | [32]  [3]  [27]  [29]  [33]  [27, 28, 34, 35]  [4, 22, 27, 28 , 34]    [34, 36]  [2, 15]  [37]  [8]  [35]  [4, 38]  [39]  [29]  [40]  [41]  [21, 42]  [22] |
| D1 | 1960s^#^ & 1970s^#^  1973 – 1981  1974; 1980^#^  1983; 1986 | Northern Ireland & UK (SSPE cases)  Australia  UK (wild-type; SSPE)  Northern Ireland | [1, 36]  [33]  [1, 27]  [36] |
| D2 | 1978 - 1996  1992; 1999 - 2002  1999 – 2000  1999; 2000  2000 | South Africa  Zambia  Ireland  UK (*unknown & Spain; Ireland)  USA (*Ireland) | [16, 34, 43]  [16, 44](unpublished data)  [45]  [8]  [39] |
| D3 | 1983; 1989 - 1992  1983 – 2001  1991 - 1994  1994  1988; 1991; 1994 - 1995  1996  1997  1997  1993^#^; 1999  1999  1999  2000; 2001; 2002; 2004  2001  2001  2002 | USA  Japan  Micronesia  Taiwan  UK  USA (*Philippines)  Denmark (*unknown)  South Africa (SSPE)  Papua New Guinea (SSPE cases)  Australia (*Japan)  Canada (*Japan)  USA (*all from Philippines)  Australia (*USA)  Canada (*unknown)  Papua New Guinea | [15, 27]  [5, 30, 46, 47]  [48]  [49]  [36, 50]  [9]  [38]  [51]  [52]  [53]  [29]  [24, 39]  [53]  [29]  (Chibo, personal communication) |
| D4 | 1978 - 1997  1988 – 1989  1989  1995 – 1997; 1999; 2000; 2001  1995; 1996; 1997; 1998; 2000; 2001  1997 – 1998  1998  1998  1998; 1999  1998 - 1999  1998 – 2000; 2002  2000; 2002  2002  2002  2002  2002  2002  2002; 2003  2003  2003 - 2004 | South Africa & Namibia  Canada  Pakistan  USA (*unknown; India & Kenya; Japan & Ethiopia; Pakistan & Kenya)  UK (*India & USA; Pakistan; India; Pakistan; Pakistan & India; Afghanistan)  India  Denmark (*unknown)  Australia (*unknown)  Netherlands (*India); Nepal  Ethiopia  Russia  Germany (*unknown; unknown)  Spain (*unknown)  UK (*Pakistan)  Canada (*India)  Australia (*unknown & Ethiopia)  Kenya  USA (*Pakistan; Lebanon)  Syria & Iran  Croatia | [16, 34, 43]  [3, 29]  [32, 54]  [2, 15, 39]  [8]  [55]  [38]  [33]  [49]  [56]  [57]  [21, 42]  [22]  [18]  [29]  (Chibo, personnel communication)  [58]  [24]  [25]  [59] |
| D5 | 1985 – 2001  1993  1993  1993 - 1994  1994; 1995; 1996  1995; 1997; 2000; 2001  1996  1997  1998  1997 – 2001  1999; 2000; 2001  2001  2001  2001 - 2002  2002  2002  2002  2002 | Japan  Micronesia (Island of Palau (*Japan))  Korea  Thailand  USA (*Japan & unknown; Japan; Japan)  UK (*unknown; Malaysia; Bangladesh; Thailand & Sth America)  Canada (*?USA)  Namibia  Australia (*unknown)  USA (*Japan & unknown)  Australia (*Thailand; unknown; Japan & Guam &Thailand & UK & unknown)  Brazil (*Japan)  Canada (*unknown & Germany & New Zealand)  Cambodia  Germany (*unknown)  UK  Guam (*unknown) & Thailand  Australia (*Thailand) | [30, 46, 47, 60]  [48]  [61]  [15]  [2, 15]  [8]  [29]  [16]  [33]  [39]  [53]  [37]  [29]  [62]  [63]  [64]  [65]  [66] |
| D6 | 1990s ^#^; 1992 – 1995  1993; 1994  1993 – 1996  1993 – 1996  1994 - 1997  mid - late 1990’s  1995 - 2001  1996; 1999  1996 – 1997  1997 – 1999  1997  1997; 1998  1993; 1999 – 2000  1997; 1998  1999  1997; 1999; 2000  1997 – 2000  1999 – 2001; 2003  2000 - 2001  2001  2003 | UK (SSPEcase); UK  UK; Spain  Spain  Germany  USA (*Spain & Germany & UK & Austria & Italy & Greece & Ukraine & Brazil)  Croatia  Russia  UK (*Greece & Germany; Poland & Ireland)  Luxembourg (*Italy & Bosnia)  Argentina  Brazil  Norway & Germany; Denmark  Netherlands (*unknown)  Russia; Poland  Uruguay  Canada (*unknown & ?USA; Netherlands; Bolivia & ?Belgium)  USA (*UK & Europe & Brazil & Turkey)  Germany  Turkey  Dominican Rep. & Haiti  USA (*Israel) | [1, 36]  [34]  [27, 28]  [4]  [2, 15]  [67]  [6]  [8]  [35]  [31, 68, 69]  [70, 71]  [38]  [72]  [4]  [73]  [29]  [39]  [21, 42, 63]  [74]  [75]  [24] |
| D7 | 1980s ^#^  1985 – 1989  1999 – 2003  1999; 2001  2000  2000; 2001  2001 - 2003  2001  2001  2002  2002; 2003  2003  2003 | UK (SSPE cases)  Australia  Germany  USA (*Sweden; Europe & unknown)  Canada (*Mexico)  Australia (*Sri Lanka; Burma)  Spain  El Salvador (*Europe)  UK (*France & Spain)  Italy & UK (*Italy)  USA (*Italy & unknown; Germany & Italy/France)  France  India | [1]  [33]  [21, 42]  [39]  [29]  [53]  [22]  [76]  [8]  [64]  [24]  [77]  [78] |
| D8 | 1994  1995; 1998; 1999; 2000; 2001  1997  1998 – 1999  1998  1999  1999  1999  2001  2002  2002  2002; 2004  2003  2003 - 2005 | UK  UK (*Pakistan; Yugoslavia & Albania & Oman; India; Bangladesh; Pakistan)  India  Ethiopia  Canada (*unknown)  Nepal  Australia (*unknown)  USA (*Italy & UK)  Australia (*India)  UK (*Lithuania & unknown)  Australia (*Bangladesh & unknown)  USA (*unknown & India; India & Bangladesh)  Spain (*unknown)  India | [56]  [8]  [55]  [56]  [29]  [49]  [53]  [39]  [53]  [18, 64]  (Chibo, personal communication)  [24]  [22]  [79] |
| D9 | 1999  2001 – 2002  2004 | Australia (*Indonesia (Is of Bali & Java))  Venezuela & Colombia (*Europe) & Indonesia  Japan (*unknown) | [53]  [65, 80]  [81] |
| d10 | 2000 - 2002 | Uganda | [82] |
| E | Mid - late 60s  1970; 1971; late 1970s  1987 | Denmark  USA (SSPE); Germany (Encephalitis); USA (wild-type & MIBE)  Canada | [32]  [27, 28 ]  [29] |
| F | 1960’s; 1979; 1994 | Spain (SSPE) | [22, 27, 28 ] |
| G1 | 1983 | USA | [3] |
| G2 | 1997  1997; 2000    1999  2000  2000  2001 | Netherlands (*Indonesia)  UK (*Indonesia; Sth Africa & Mexico & Australia)  Indonesia & Malaysia  USA (*unknown)  Australia (*Malaysia & unknown)  Germany (*unknown) | [83]  [8]  [84]  [39]  [53]  [21] |
| G3 | 1999  2002 | East Timor & Australia (*unknown & East Timor)  Indonesia | [85]  [86] |
| H1 | 1993 - 1994  1993  1995 – 1999; 2001  1996  1997  1997  1997 - 2001  1998  2000  2000 - 2001  2000 – 2001  2000 - 2001  2000; 2001; 2002  2001  2001  2002  2003; 2004  2003; 2004 | China  Australia (*unknown)  China  UK  Netherlands (*China)  New Zealand (*unknown)  USA (*China & unknown & Korea)  Denmark (*Thailand)  UK (*USA )  Australia (*unknown & Japan)  Korea  Canada (*Korea & Singapore)  Japan (*unknown; unknown; unknown)  Germany (*Mongolia)  Spain (*China)  Australia (*unknown) & Mongolia  Rep. of Marshall Islands & Chile (*Japan) & Mexico (*unknown); Mexico (*unknown)  USA (*Rep. Marshall Islands & unknown; China) | [10]  [33]  [87-89]  [89]  [49]  [90]  [39]  [38]  [8]  [53]  [91]  [29]  [92, 93]  [42, 63]  [22]  (Chibo, personal communication)  [65, 94]  [24] |
| H2 | 1994  1997; 2000; 2001  1998  2002 | China  USA (*Vietnam; unknown; unknown)  Vietnam  Australia (*unknown) | [10]  [39]  [88]  (Chibo, personal communication) |

**References:**
